# Supplementary material for: PGE2 and Poloxamer Synperonic F108 Enhance Transduction of Human HSPCs with a β-Globin Lentiviral Vector
Source: Mol Ther Methods Clin Dev. 2019 Apr 4;13:390–8. doi: 10.1016/j.omtm.2019.03.005 (PMC6477655; doi:10.1016/j.omtm.2019.03.005)
Supplement: Document S1. Figures S1–S3 [file mmc1.pdf]

OMTM, Volume *13*

Supplemental Information

PGE2 and Poloxamer Synperonic F108 Enhance Transduction of Human HSPCs with a β-Globin Lentiviral Vector

Katelyn E. Masiuk, Ruixue Zhang, Kyle Osborne, Roger P. Hollis, Beatriz Campo-Fernandez, and Donald B. Kohn

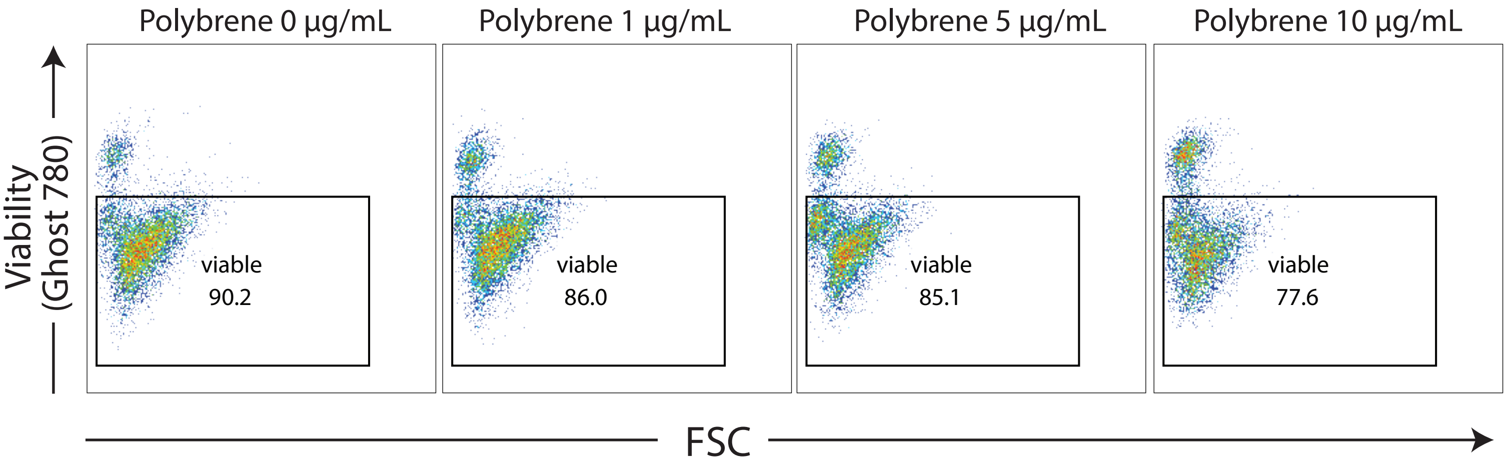

Figure S1: Dose-dependent toxicity of polybrene in LV transduction cultures

G-CSF mPB CD34+ cells were transduced with Globe1-AS3 in the presence of PGE2/poloxamer F-108 and increasing doses of polybrene. FACS plots show viability of cells measured 24 hours post-transduction

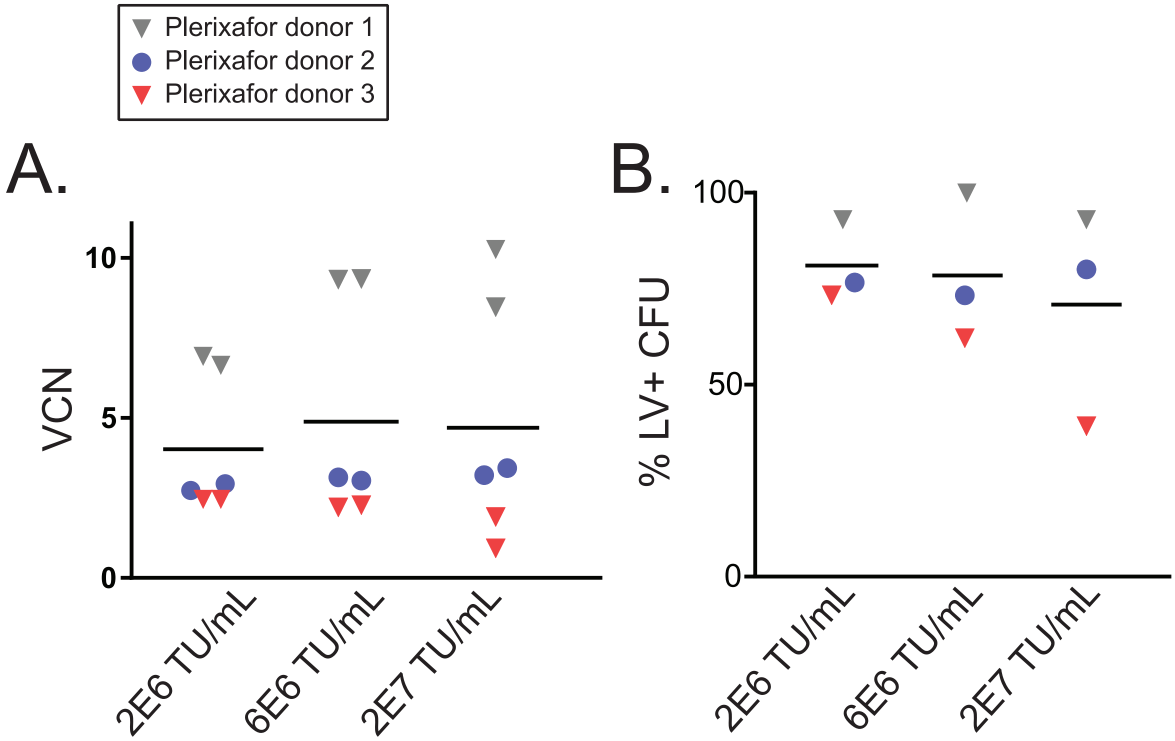

Figure S2: Transduction of Plerixafor-mobilized CD34+ cells with LV Globe1-AS3

A. VCN in 12 day myeloid differentiated cultures for Plerixafor mPB CD34+ cells transduced with transduction enhancers at 3 different LV doses. Data represent measurements from 2 replicate culture wells for 3 independent mPB CD34+ donors (each marked with a distinct color/symbol).
B. Percentage of individual colonies containing integrated viral copies for Plerixafor mPB CD34+ cells transduced with transduction enhancers at 3 different LV doses. A positive colony was defined as VCN>0.5. Data represent a single percentage (calculated from 30 analyzed colonies) for each LV dose in each of 3 independent CD34+ donors.

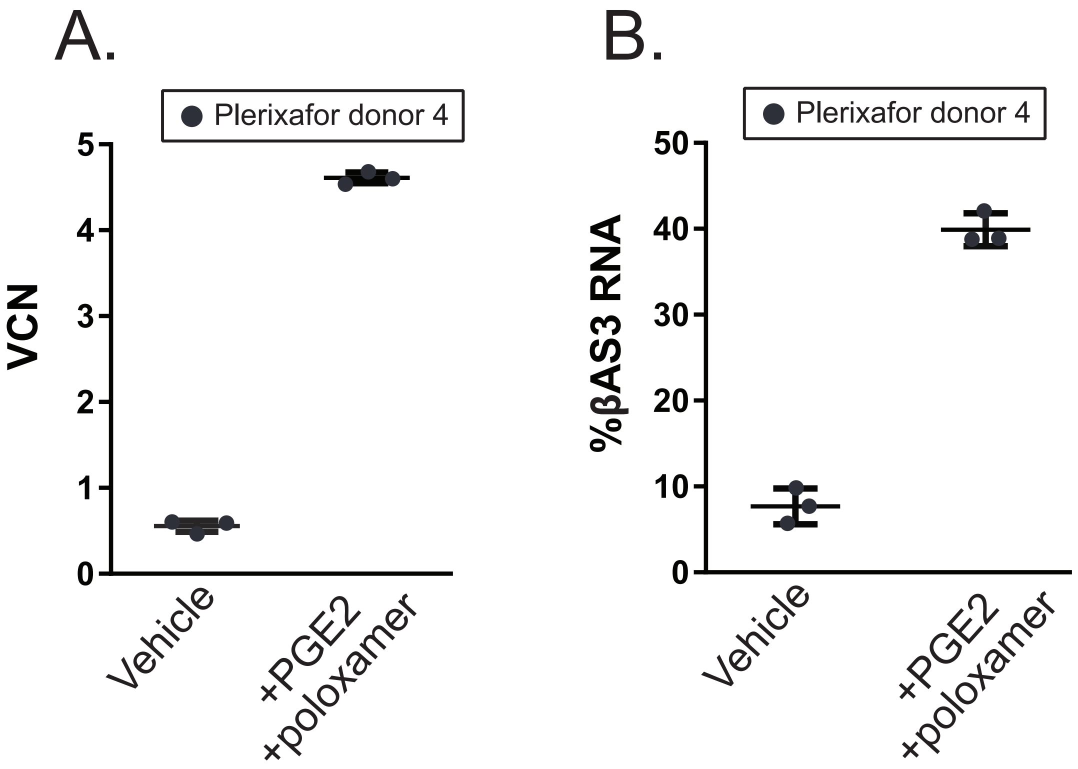

Figure S3: Correlation between enhanced VCN and enhanced βAS3 transgene expression

A. VCN in 12 day erythroid differentiated cultures for Plerixafor mPB CD34+ cells transduced with vehicle control or transduction enhancers at $2x10^6$ TU/mL. Data represent mean ± SD of 3 replicate culture wells from one CD34+ donor.

B. Corresponding percentage of viral βAS3 RNA (as a percentage of total β-globin RNA) measured in erythroid differentiated cells. Data represent mean ± SD of 3 replicate culture wells from one CD34+ donor.

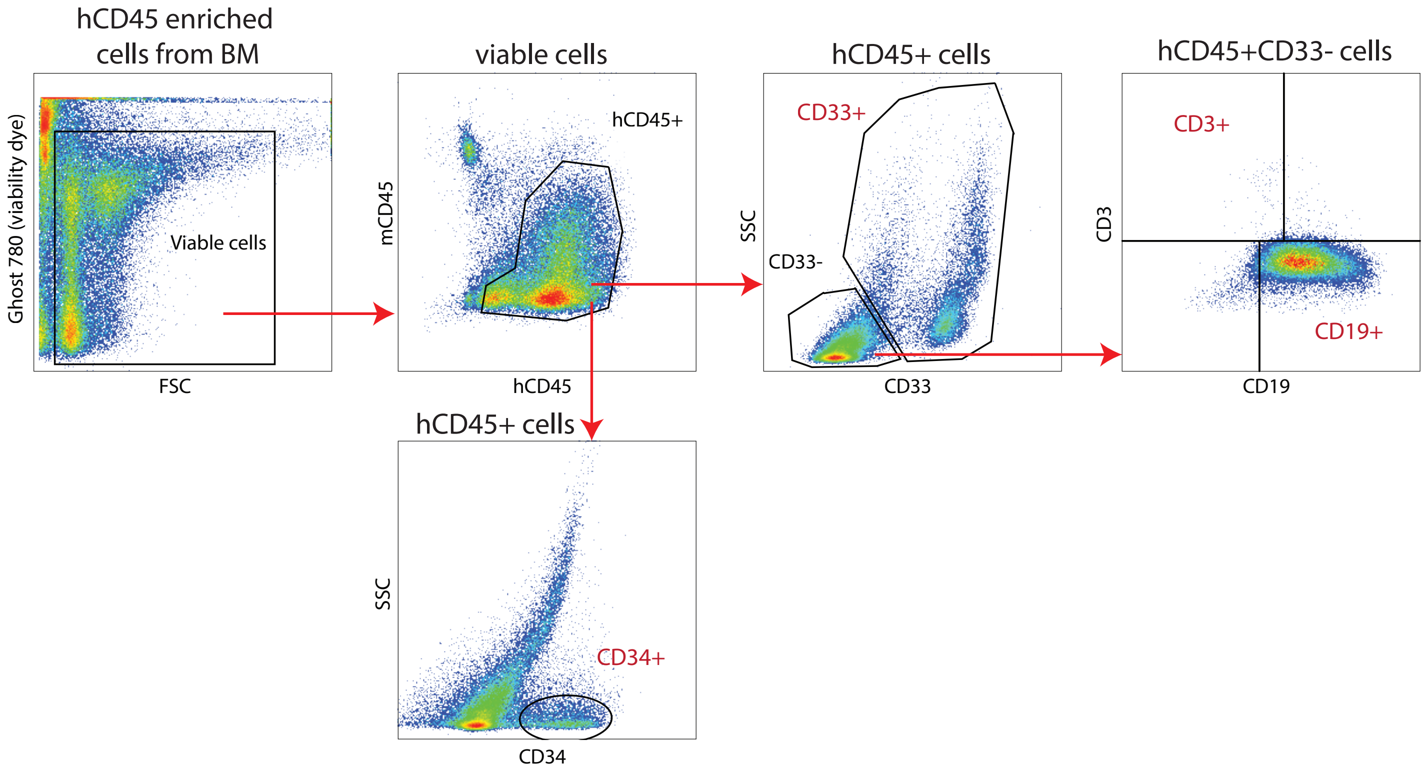

Figure S4: Determination of lineage distribution in NSG xenografts

Human CD45+ cells were magnetically enriched from the BM of engrafted NSG mice. Plots show flow cytometry gating strategy used to determine the relative contribution of each lineage to total engrafted hCD45+ cells. Lineages analyzed are marked in red and include: CD34+, CD33+, CD3+, and CD19+.
