# Supplementary material for: PGE2 and Poloxamer Synperonic F108 Enhance Transduction of Human HSPCs with a β-Globin Lentiviral Vector
Source: Mol Ther Methods Clin Dev. 2019 Apr 4;13:390–8. doi: 10.1016/j.omtm.2019.03.005 (PMC6477655; doi:10.1016/j.omtm.2019.03.005)
Supplement: Document S2. Article plus Supplemental Information [file mmc2.pdf]

Molecular Therapy
Methods & Clinical Development
Original Article

PGE2 and Poloxamer Synperonic F108 Enhance Transduction of Human HSPCs with a β-Globin Lentiviral Vector

Katelyn E. Masiuk,[1] Ruixue Zhang,[1] Kyle Osborne,[1] Roger P. Hollis,[1] Beatriz Campo-Fernandez,[1] and Donald B. Kohn[1,2,3]

[1]Department of Microbiology, Immunology & Molecular Genetics, University of California, Los Angeles, Los Angeles, CA 90095, USA; [2]Department of Pediatrics, David Geffen School of Medicine, University of California, Los Angeles, Los Angeles, CA 90095, USA; [3]Department of Molecular & Medical Pharmacology, David Geffen School of Medicine, University of California, Los Angeles, Los Angeles, CA 90095, USA

Lentiviral vector (LV)-based hematopoietic stem and progenitor cell (HSPC) gene therapy is becoming a promising alternative to allogeneic stem cell transplantation for curing genetic diseases. Clinical trials are currently underway to treat sickle cell disease using LVs expressing designed anti-sickling globin genes. However, because of the large size and complexity of the human β-globin gene, LV products often have low titers and transduction efficiency, requiring large amounts to treat a single patient. Furthermore, transduction of patient HSPCs often fails to achieve a sufficiently high vector copy number (VCN) and transgene expression for clinical benefit. We therefore investigated the combination of two compounds (PGE2 and poloxamer synperonic F108) to enhance transduction of HSPCs with a clinical-scale preparation of Lenti/G-AS3-FB. Here, we found that transduction enhancers increased the *in vitro* VCN of bulk myeloid cultures ~10-fold while using a 10-fold lower LV dose. This was accompanied by an increased percentage of transduced colony-forming units. Importantly, analysis of immune-deficient NSG xenografts revealed that the combination of PGE2/synperonic F108 increased LV gene transfer in a primitive HSC population, with no effects on lineage distribution or engraftment. The use of transduction enhancers may greatly improve efficacy for LV-based HSPC gene therapy.

INTRODUCTION

Sickle cell disease (SCD) is the most prevalent monogenic blood disorder, affecting 100,000 people in the United States and millions worldwide.[1,2] SCD is caused by a point mutation in the β-globin gene that leads to hemoglobin polymerization and sickling of red blood cells under conditions of low oxygen tension. Sickled red blood cells lead to a number of vascular complications, such as pain crises, stroke, and organ damage, which ultimately result in significant morbidity and early mortality.[3]

Currently available medical treatments for SCD are aimed at managing disease burden, but the only curative option is allogeneic hematopoietic stem cell (HSC) transplantation. However, allogeneic HSC transplantation is unavailable to most patients because of the lack of an immunologically matched donor. In recent years, autologous HSC transplant with gene therapy has emerged as a promising alternative that allows patients to serve as their own HSC donor. In this approach, hematopoietic stem and progenitor cells (HSPCs) are collected from a patient, modified *ex vivo* using lentiviral vectors (LVs) to express an anti-sickling globin transgene, and transplanted back into the patient to engraft the bone marrow (BM) and provide a durable source of healthy, non-sickled red blood cells.

Many successful gene therapies for other genetic blood disorders have used relatively simple LVs with small genomes to drive high levels of a transgene product from a constitutively active, strong promoter.[4] In contrast, current LV candidates for SCD are more complex and utilize large elements of the endogenous β-globin locus control region (LCR) to drive erythroid-specific expression of anti-sickling globin transgenes in mature erythrocytes.[5,6] The large size and complex nature of these LVs has led to a number of hurdles for clinical translation. Globin LVs exhibit poor titers and require large production volumes of the guanosine monophosphate (GMP)-grade LV, which is both expensive and technically challenging to produce, to treat a single patient. Additionally, gene transfer of globin LVs to primitive HSCs has been poor, often failing to achieve sufficiently high vector copy numbers and transgene expression to correct the disease.[7] Thus, new methods to improve transduction efficiency of HSCs are critical to the future success of gene therapy for SCD, using these vectors.

Recently, Heffner et al.[8] performed a small-molecule screen to identify compounds that enhance LV transduction of HSPCs and identified prostaglandin E2 (PGE2) as a leading candidate. They further showed that PGE2 can increase vector copy numbers (VCNs) of

Received 8 March 2019; accepted 26 March 2019;
https://doi.org/10.1016/j.omtm.2019.03.005.

Correspondence: Donald B. Kohn, MD, Department of Microbiology, Immunology & Molecular Genetics, University of California, Los Angeles, Los Angeles, CA 90095, USA.
E-mail: dkohn1@mednet.ucla.edu

 © 2019 The Author(s).
This is an open access article under the CC BY license (http://creativecommons.org/licenses/by/4.0/).

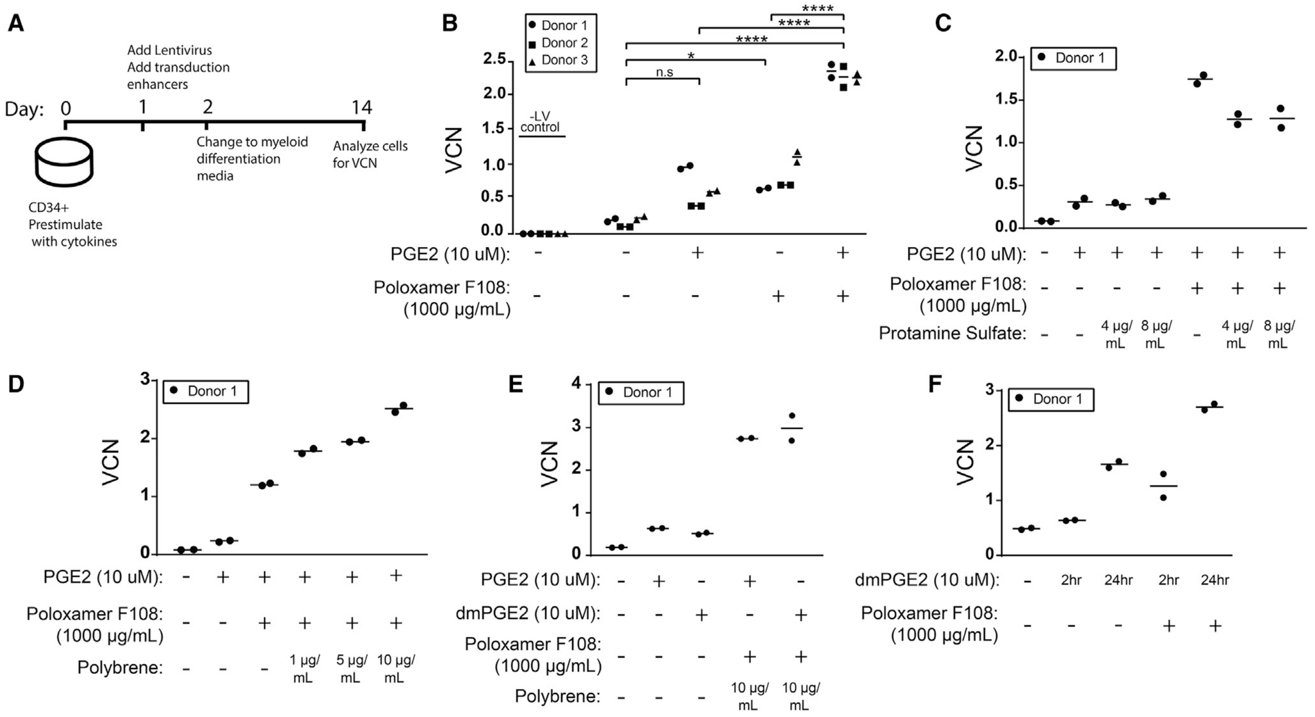

Figure 1. Optimization of Transduction Enhancers for Increased Gene Transfer to G-CSF mPB CD34+ Cells

(A) Experimental set-up for determination of VCN: G-CSF mPB CD34+ cells were pre-stimulated with cytokines for 24 h. Lenti/G-AS3-FB was added, with or without transduction enhancers, for an additional 24 h. The following day, cells were washed and transferred into an *in vitro* myeloid differentiation culture. VCN was analyzed after 12 days of culture. (B) G-CSF mPB CD34+ HSPCs were transduced in the presence of different drug combinations with Lenti/G-AS3-FB at 2×10^7 TU/mL. Data represent the mean of two replicate culture wells for three independent mPB CD34+ donors. (n = 3/arm; one-way ANOVA with Bonferroni's multiple comparison, n.s. not significant, $^*p \le 0.05$, $^{****}p \le 0.0001$). (C–E) G-CSF mPB CD34+ HSPCs were transduced in the presence of different drug combinations (C: protamine sulface; D: polybrene; and E: dmPGE2) with Lenti/G-AS3-FB at 2×10^7 TU/mL (bars represent the mean, n = 2 replicate wells/condition). (F) G-CSF mPB CD34+ HSPCs were pre-treated with a pulse of dmPGE2 for 2 h, washed, and transduced with LV, or treated with PGE2 for 24 h, at the same time as the addition of LV (bars represent the mean, n = 2 replicate wells/condition).

short-term CD34+ hematopoietic progenitor cells by ~2-fold with a simple GFP LV; however, the VCN increase achieved in long-term HSCs (LT-HSCs) from NOD-scid IL2Rg^{null} (NSG) xenografts was more modest (1.5-fold).

Additionally, Hauber et al.[9] reported that a combination of poloxamer synperonic F108 and polybrene (commercially available as LentiBoost) enhanced transduction of short-term CD34+ hematopoietic progenitor cells by ~2.5-fold with a simple GFP LV. Transduction enhancement of LT-HSCs with LentiBoost in NSG xenografts was not explored.

Here, using a clinical preparation of a globin LV (Lenti/G-AS3-FB), we found that the combination of PGE2 and poloxamer synperonic F108 markedly enhanced gene transfer (~10-fold) in CD34+ HSPCs. These effects were reproducible among CD34+ HSPCs from multiple donors mobilized by either granulocyte colony-stimulating factor (G-CSF) or plerixafor. Importantly, transduction enhancement (~6-fold) was evident in NSG xenografts *in vivo*, indicating that these compounds effectively target primitive CD34+ cells capable of 15-week engraftment. Collectively, these results suggest that the addition of transduction enhancers to current gene therapy trials for SCD may overcome the limiting obstacles and achieve sufficiently high VCNs for clinical benefit.

RESULTS

PGE2 and Poloxamer Synperonic F108 Increase Gene Transfer of a Globin LV in CD34+ HSPCs

We first explored the use of PGE2 and poloxamer synperonic F108 to enhance transduction of G-CSF mobilized peripheral blood (mPB) CD34+ HSPC with a clinical preparation of Lenti/G-AS3-FB (Figure 1A). Similar to previously reported work using simple GFP LVs,[8,9] we found that both PGE2 and poloxamer synperonic F108 used alone enhanced transduction with a complex globin LV. Strikingly, the combination of the two compounds showed synergistic effects, enhancing LV transduction ~10-fold (Figure 1B). We further explored the combination with the commonly used transduction enhancers protamine sulfate and polybrene. The addition of protamine sulfate did not further enhance transduction (Figure 1C), whereas the addition of polybrene showed a dose-dependent increase in VCN (Figure 1D). However, the addition of polybrene also exhibited a dose-dependent toxicity (Figure S1). We additionally

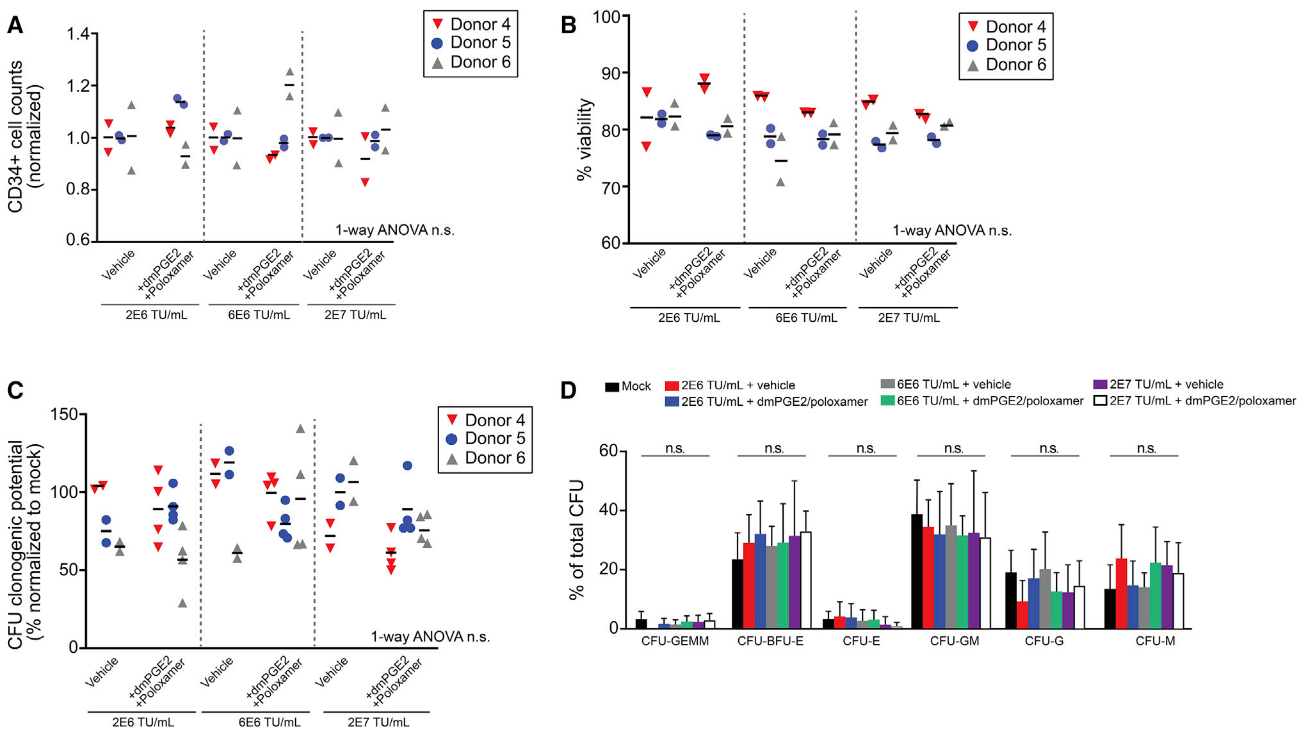

Figure 2. Transduction Enhancers Do Not Affect the Viability or Clonogenic Potential of G-CSF mPB CD34+ Cells

(A) CD34+ cell counts at 24 h following transduction with and without transduction enhancers at three different LV doses. Data represent measurements from two replicate culture wells for three independent mPB CD34+ donors (each marked with a distinct color/symbol). For each combination of donor/LV dose, the cell count was normalized to vehicle control (bars represent the mean for each donor; n = 3/arm; one-way ANOVA; n.s. not significant). (B) Percentage of viable cells at 24 h following transduction determined by flow cytometry viability staining analysis. Data represent measurements from two replicate culture wells for three independent mPB CD34+ donors (bars represent the mean for each donor; n = 3/arm; one-way ANOVA; n.s. not significant). (C) Clonogenic potential (percentage of colonies formed of total cells plated) of transduced CD34+ cells. Data represent measurements from two to four replicate CFU cultures for three independent mPB CD34+ donors. Data for each donor are normalized to the mean clonogenic potential of four replicate non-transduced "mock" control wells for that donor (bars represent mean for each donor; n = 3/arm, one-way ANOVA, n.s. not significant). (D) CFU lineage distribution for transduced CD34+ cells. CFUs were scored in the following categories: CFU-GEMM (CFU-granulocyte/erythroid/macrophage/megakaryocyte), BFU-E (burst-forming unit-erythroid), CFU-E (CFU-erythroid), CFU-GM (CFU-granulocyte/macrophage), CFU-G (CFU-granulocyte), and CFU-GM (CFU-macrophage). Data show the frequency of each individual colony type as a percentage of total colonies. Data represent measurements from two to four replicate CFU cultures for three independent mPB CD34+ donors (bars represent the mean ± SD; one-way ANOVA, n.s. not significant).

compared the transduction-enhancing effects of PGE2 to the more stable PGE2 analog 16,16-dimethyl-PGE2 (dmPGE2) which has been used clinically[10] and is available in GMP formulations. Here, we found the two compounds to be interchangeable in their effects on transduction (Figure 1E). Based on current clinical protocols for expanding cord blood units, which use a 2-h pulse exposure of PGE2 to promote HSC engraftment,[10] we evaluated the transduction enhancement effects of 2-h PGE2 pulse exposure prior to addition of the LV. Here, we observed that a 2-h PGE2 pulse exposure modestly enhanced transduction, but greater transduction enhancement was achieved with a 24-h PGE2 exposure (Figure 1F).

PGE2 and Poloxamer Synperonic F108 Mediate Increased Transduction in Multiple HSPC Donors Mobilized with G-CSF and Plerixafor

We next evaluated the combination of 10 μM dmPGE2 and 1 mg/mL poloxamer synperonic F108 in G-CSF mobilized PB CD34+ cells from three different healthy donor cell lots. Cells were transduced with a clinical preparation of Lenti/G-AS3-FB at a range of concentrations (2×10^6 transduction units [TU]/mL–2×10^7 TU/mL) in the presence of dmPGE2/poloxamer synperonic F108 or vehicle control. We first assessed any potential toxicity of these compounds. Here, we found that the addition of dmPGE2/poloxamer synperonic F108 did not alter viable CD34+ cell counts or the percentage of viable cells (Figures 2A and 2B). Methylcellulose cultures plated after transduction revealed no differences in clonogenic potential nor lineage differentiation (Figures 2C and 2D).

VCN analysis of bulk myeloid cultures 12 days after transduction revealed a consistent ~10-fold increase in VCN, even at an LV dose of 2×10^6 TU/mL (10-fold lower than the current clinical protocol dose) (Figure 3A). In order to determine if the increased gene transfer in bulk cultures reflected increased integration to a small number of cells or an increase in the percentage of transduced cells, the VCN was determined for individual colony-forming units (CFUs) from methylcellulose cultures. Both myeloid and erythroid colonies transduced

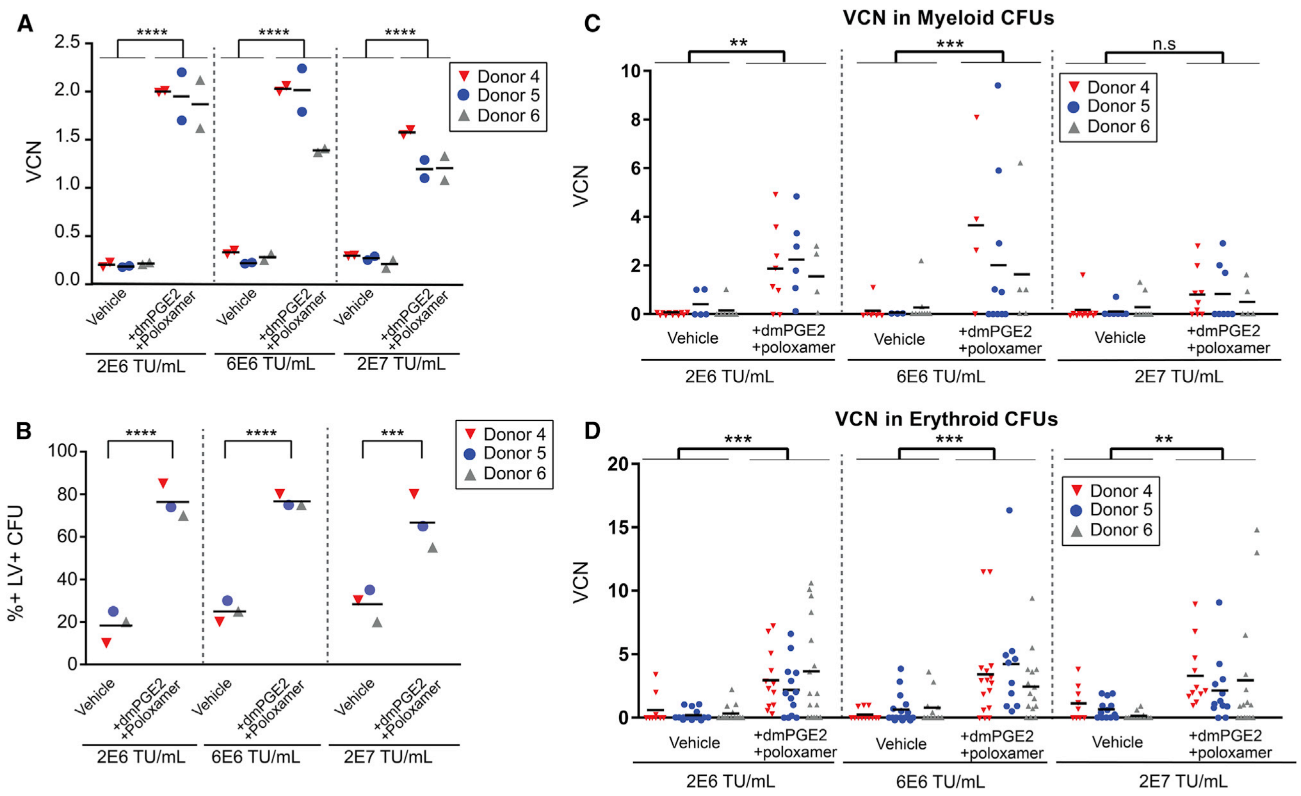

Figure 3. Transduction Enhancers Improve Gene Transfer in G-CSF mPB CD34+ Cells

(A) VCN in 12-day myeloid differentiated cultures is shown for G-CSF mPB CD34+ cells transduced with and without transduction enhancers at three different LV doses. Data represent measurements from two replicate culture wells for three independent mPB CD34+ donors (bars represent the mean for each donor; n = 3; one-way ANOVA with Bonferroni's multiple comparison; ****$p \leq 0.0001$). (B) Percentage of individual colonies containing integrated viral copies is shown for G-CSF mPB CD34+ cells transduced with and without transduction enhancers at three different LV doses. A positive colony was defined as VCN > 0.5. Data represent a single percentage (calculated from 20 analyzed colonies) for each LV dose/transduction condition for three independent mPB CD34+ donors (bars represent mean; n = 3; one-way ANOVA with Bonferroni's multiple comparison, ***$p \leq 0.001$, ****$p \leq 0.0001$). (C) VCN in individual myeloid CFU colonies. Data represent VCN measured in 3–11 colonies per donor/transduction condition/LV dose (bar represents mean VCN for each donor/condition; n = 3; one-way ANOVA with Bonferroni's multiple comparison; **$p \leq 0.01$, ***$p \leq 0.001$, n.s. not significant). (D) VCN in individual erythroid CFU colonies. Data represent VCN measured in 9–17 colonies per donor/transduction condition/LV dose (bar represents the mean for each donor/condition; n = 3; one-way ANOVA with Bonferroni's multiple comparison; **$p \leq 0.01$, ***$p \leq 0.001$).

in the presence of transduction enhancers showed a dramatic increase in the percentage of transduced cells (Figure 3B) and in the average VCN in transduced cells (Figures 3C and 3D).

Although G-CSF mobilization represents the standard collection regimen for isolating HSCs from healthy adult donors, the use of G-CSF is contraindicated in patients with SCD because of its potential to induce a sickle cell crisis.[11] Recently, it has been demonstrated that plerixafor can achieve safe and successful mobilization of patients with SCD and produces higher CD34+ cell doses than those that can historically be achieved using traditional BM aspiration.[12] As plerixafor-mobilized CD34+ will likely be the preferred source of HSPCs for SCD gene therapy, we confirmed that transduction enhancers show similar efficacy in this cell type. Transduction of three different cell lots of plerixafor-mobilized CD34+ cells with Lenti/G-AS3-FB in the presence of dmPGE2 and poloxamer synperonic F108 achieved a high VCN and percentage of PCR+ colonies (Figure S2). Furthermore, erythroid differentiation of transduced cells showed enhanced expression of the LV transgene RNA (βAS3 globin) (Figure S3), confirming that transduction enhancers facilitate increased net transgene expression in the population of modified cells.

Transduction Enhancers Facilitate Improved Gene Transfer in HSCs Capable of Long-Term Engraftment in NSG Mice

We next evaluated the effect of PGE2/poloxamer synperonic F108 treatment on *in vivo* hematopoiesis, using NSG xenografts. G-CSF-mobilized CD34+ HSPCs from three independent donor cell lots were transduced with Lenti/G-AS3-FB at 2 × 10^6 TU/mL in the presence of no culture additives, vehicle controls (0.1% DMSO and 1% H_2O), or PGE2/poloxamer synperonic F108 and injected into sublethally irradiated NSG mice. At 6 weeks after the transplant, PB engraftment analyses revealed no differences among the arms, suggesting that transduction enhancers do not impair

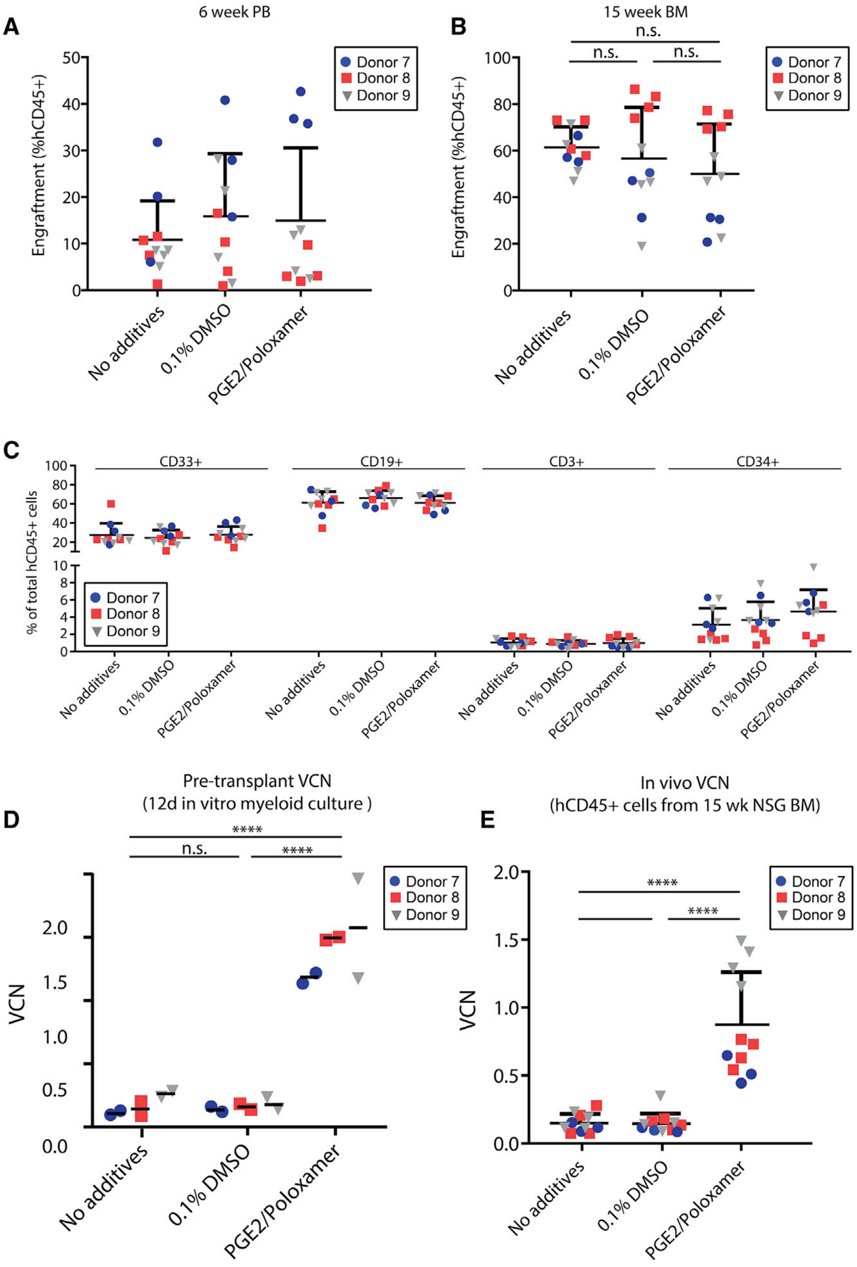

Figure 4. Transduction Enhancers Achieve High Copy Number in NSG Xenografts

(A) PB engraftment levels at 6 weeks after transplant. Engraftment was defined as the percentage of human CD45$^+$ cells of total CD45$^+$ cells (mCD45$^+$ hCD45). G-CSF mPB CD34$^+$ cells from three independent CD34$^+$ donors (each marked with a distinct color/symbol) were transplanted into 11 different NSG mice per condition (mean ± SD; n = 11; one-way ANOVA; n.s. not significant). (B) Engraftment levels in the BM at 15 weeks after transplant (mean ± SD; n = 11; one-way ANOVA with Tukey's multiple comparisons; n.s. not significant). (C) Lineage distribution of engrafted hCD45$^+$ cells. Lineages are represented as a percentage of total hCD45$^+$ cells (mean ± SD; one-way ANOVA; n.s. for all lineages). (D) Pre-transplant VCN measured for each donor in 12 day myeloid culture assay (bars represent mean of two replicate wells for each donor/condition; n = 3; one-way ANOVA with Tukey's multiple comparison; ****$p \leq 0.0001$, n.s. not significant). (E) *In vivo* VCN measured in hCD45$^+$ cells engrafted in NSG BM (mean ± SD; one-way ANOVA; with Tukey's multiple comparisons; ****$p \leq 0.0001$, n.s. not significant).

early hematopoiesis from engrafting progenitor cells (Figure 4A). Similarly, no differences among groups were observed for longer term (15 week) BM engraftment (Figure 4B) and lineage distribution of engrafted cells (Figures 4C and S4), suggesting no adverse effects of transduction enhancers on HSPC function. *In vitro* copy number analysis of transplanted cells (analyzed by short-term myeloid culture) revealed a typical ~10-fold increase in VCN in cells treated with PGE2/poloxamer synperonic F108 (Figure 4D). *In vivo*, engrafted hCD45$^+$ transduced in the presence of transduction enhancers showed a ~6-fold increase in VCN, confirming that PGE2/poloxamer synperonic F108 enhances transduction of a primitive HSC population capable of longer-term NSG engraftment (Figure 4E).

DISCUSSION

While gene therapy for SCD has made remarkable progress, poor gene transfer of globin LV remains an important hurdle for successful clinical translation. A number of transduction enhancers have been reported to improve LV transduction of HSPCs, including Vectofusin-1,[13] rapamycin,[14] cyclosporine A,[15] UM171,[16] staurosporine,[17] and cyclosporine H.[18] In our hands, many transduction-enhancing compounds that show promising results using simple GFP LVs have had minimal results in transduction enhancement of globin LVs. This may highlight the relative difficulty of using globin LVs, compared with simple GFP LVs; transduction of HSPCs with globin LVs often saturates at low VCNs and does not increase at higher LV doses. Limits in the transduction efficiency of globin LV may be due to their large size[19] or complex design. Notably, our experiments here have shown that the combination of PGE2 and poloxamer synperonic F108 markedly enhances HSPC transduction with globin LVs to levels which we anticipate to be clinically efficacious.

Although the magnitude of transduction enhancement that we observed with each PGE2 and poloxamer synperonic F108 alone was consistent with prior reports,[8,9] we observed an additive and potentially synergistic transduction enhancement of these compounds when used in combination. It is possible that each compound

may enhance viral transduction at different steps of the viral life cycle. Poloxamers are amphiphilic polymers that have been shown to fluidize membranes,[20] increase lipid exchange, and enhance transmembrane transport.[21,22] Thus, a possible mechanism of action may involve enhancing the interaction between viral particles and the host cell membrane.

When transducing HSPCs with PGE2, Zonari et al.[23] detected a marked increase in late RT copies within 6 h after transduction, suggesting that PGE2 mediates transduction enhancement prior to nuclear entry and integration. Additional experiments by Zonari et al. suggested that PGE2 does not work through cyclophilin A-mediated uncoating, but it does affect endocytosis, as no improvement in VCN is seen with non-endocytosis-dependent envelopes. Additionally, Heffner et al.[8] found no effect of PGE2 on viral fusion, using a VPR-beta-lactamase assay, which implies that the enhancement occurs after fusion but during the endocytosis phase of viral transduction. Future work to elucidate the mechanisms of these compounds may further aid in improving LV transduction methods.

Unexpectedly, $CD34^+$ cells transduced with PGE2/poloxamer synperonic F108 showed a dose-dependent decrease in VCN when increasing LV dose. This is in contrast to the modest dose-dependent increase in VCN observed in the absence of transduction enhancers. One possibility that could explain this finding is that high LV doses of 2×10^7 TU/mL in the presence of PGE2/poloxamer synperonic F108 increases transduction to a toxic level, with highly transduced cells being selectively eliminated. Although we cannot rule out this possibility, we note that high VCN colonies were still detectable at the lower (2×10^6 TU/mL) LV dose and that no clear relationship was observed with cell counts, viability, or clonogenic potential and LV dose. An additional speculative possibility is that there is an optimal ratio for poloxamer molecules and LV particles, where high levels of LV particles may become detrimental to transduction. Although this mechanism remains to be explored, investigators using this combination of compounds may need to titrate individual LV dose. Importantly, using a lower LV dose to achieve higher VCN may be highly advantageous for clinical gene therapy, because of the vast expense and time required to produce and validate each lot of GMP-grade LV.

Interestingly, we observed marked enhancement of HSPC transduction *in vitro*, but this effect was somewhat diminished in NSG xenografts. This is consistent with results observed by Heffner et al.,[8] using PGE2 alone, and suggests that PGE2/poloxamer synperonic F108 may preferentially enhance transduction of short-term progenitor cells with more modest effects in the LT-HSC compartment. Nonetheless, we predict that if a 6-fold increase in gene transfer to primitive HSCs could be achieved in a clinical setting, it would be sufficient to achieve a therapeutic transgene expression level.

An important consideration surrounding this work is defining a benchmark for a drug product (DP) VCN *in vitro*, which may be clinically efficacious after transplant. The first promising case of SCD gene therapy was reported in the HGB-205 trial with LentiGlobin BB305.[24] A 12-year-old patient, who had been on prophylactic red-cell transfusions for 4 years prior to treatment, received a $CD34^+$ cell product with an average VCN of 1.1. After 15 months of follow-up, the patient showed stable gene marking in the PB (VCN ~2) and expressed therapeutic anti-sickling globin in 48% of all globin tetramers. Clinical outcomes and analysis of SCD-related biologic parameters indicated stable correction of the disease, suggesting that SCD gene therapy can be successful when high VCN and transgene expression are achieved.

In contrast, subsequent SCD patients treated in the HGB-206 study showed less efficacious results; PB levels of the LentiGlobin BB305 vector were low (median = 0.08; range, 0.05–0.13) in all treated subjects, with no evidence of clinical benefit.[7] Similarly, a cohort of patients with beta thalassemia treated with LentiGlobin BB305 in the HGB-204 study showed low PB VCN (median = 0.3; range, 0.1–0.9).[25] In both of these studies, the *in vitro* drug product VCN measured prior to transplant was much higher (HGB-205: median = 0.6; range, 0.3–1.3; HGB-204: median = 0.7; range, 0.3–1.5), representing a 2- to 8-fold drop-off after transplant.

The observed drop between the measured drug product VCN *in vitro* and the post-engraftment PB VCN *in vivo* is likely multifactorial. One reason for this observed effect is the limitation of *in vitro* assays, which predominantly measure gene transfer to short-term erythroid or myeloid progenitor cells. As progenitor cells are more easily transduced than LT-HSCs, these assays often overestimate the true *in vivo* VCN determined by the level of gene transfer into LT-HSCs. This effect is well understood in the field and can be observed here in our NSG xenograft experiments. Therefore, although an ideal target for *in vivo* VCN may be around 2 copies/cell, using *in vitro* assays to predict a target drug product VCN for an individual patient may be challenging in practice.

An additional factor that may affect the observed drop between *in vitro* and *in vivo* VCN (in a clinical transplant setting) is the competition between non-modified endogenous HSCs and modified transplanted HSCs. Thus, the number of gene-modified cells retained *in vivo* can be highly dependent on the quality of the graft and the effectiveness of the conditioning regimen. The problem of low-quality HSCs may be of particular importance in SCD because of the sub-optimal $CD34^+$ cell number obtained from BM collection and reduced quality of HSCs obtained from an inflammatory BM environment.[26] Transfusion lead-in and plerixafor mobilization protocols have been explored to improve the quality and quantity of $CD34^+$ HSPCs collected[12,27] and monitoring of plasma busulfan concentrations with dose adjustments has been explored to optimize myeloablation.[25]

Although improvements in HSCs quality and engraftment may mitigate the observed decline in VCN transplant, clinical data suggest that improvements in pre-transplant *in vitro* VCNs are also required. In support of this, early clinical results from the HGB-206 study describing a new protocol with a proprietary method of lentiviral

transduction have reported both enhanced drug product VCNs (median = 4.0; range, 2.8 – 5.6) and early *in vivo* PB VCNs (range 1.4–2.9).[27]

Although we can only cautiously compare the reported *in vitro* drug product VCNs from the HGB-206 study to our own experiments (because of differences in LV construct, GMP manufacturing process, and protocols for measuring *in vitro* VCNs), our transduction protocol using PGE2/poloxamer synperonic F108 in plerixafor-mobilized $CD34^+$ donors yielded an *in vitro* drug product VCN of 2.5–6.8. Therefore, we anticipate that a historic 2-fold decrease in VCN after transplant would still maintain *in vivo* VCNs in a therapeutically efficacious range. As more patients are treated, we anticipate that we can better characterize the correlation between *in vitro* and *in vivo* VCNs and may be able to further refine targets for the *in vitro* VCN prior to transplant.

In summary, the use of PGE2/poloxamer synperonic F108 as transduction enhancers provides a promising strategy to overcome current limitations in gene therapy for SCD and may further support the clinical translation of novel LV-based HSPC gene therapies for genetic blood cell disorders of different origins.

MATERIALS AND METHODS

Transduction Enhancers

Poloxamer synperonic F108 (Kolliphor P338; BASF, Ludwigshafen, Germany) was prepared in a stock solution of 100 mg/mL by dissolving it in sterile water overnight and filtering it through a 0.22 μm filter. The poloxamer synperonic F108 stock solution was stored at 4°C. PGE2 and dmPGE2 (Cayman Chemicals, Ann Arbor, MI, USA) were dissolved separately in DMSO to make 10 mM stock solutions and stored as single-use aliquots at −80°C.

Lentiviral Transduction

$CD34^+$ cells from healthy donors mobilized with G-CSF or plerixafor were purchased from Hemacare (Van Nuys, CA, USA) or StemExpress (Folsom, CA, USA). The cells were plated at a density of 5×10^5–1×10^6 cells/mL in X-Vivo-15 (Lonza, Basel, Switzerland) with 1 × L-glutamine-penicillin-streptomycin (L-Glut-Pen-Strep), Gemini BioProducts, West Sacramento, CA, USA), 50 ng/mL SCF, 50 ng/mL TPO, and 50 ng/mL Flt3L (PeproTech, Rocky Hill, NJ, USA) and cultured in 5% CO_2, 37°C, and a humidified atmosphere throughout. Twenty-four hours after cytokine pre-stimulation, the cells were transduced by adding Lenti/G-AS3-FB (Lentigen, Gaithersburg, MD, USA) and transduction enhancers or vehicle control directly to the cells. Twenty-four hours after transduction, the cells were collected, washed, and used for the downstream applications described below.

In Vitro Myeloid Differentiation Cultures

Transduced $CD34^+$ cells were cultured in Basal BM Medium (BBMM; Iscove's Modified Dulbecco's Medium [IMDM]; Life Technologies, Grand Island, NY), 1 × L-glutamine-penicillin-streptomycin (LGlut-Pen-Strep), 20% fetal bovine serum [FBS], 0.52% BSA) with cytokines (5 ng/mL IL-3, 10 ng/mL IL-6, 25 ng/mL hSCF, [PeproTech]) at 37°C, 5% CO_2. Cells were split every 2–3 days and supplemented with fresh BBMM plus cytokines. After 12 days of culture, the cells were collected, and genomic DNA was extracted, using the Purelink Genomic DNA Mini Kit (Invitrogen, Carlsbad, CA, USA).

In Vitro Erythroid Differentiation

Transduced $CD34^+$ cells were transferred into erythroid culture. The *in vitro* erythroid differentiation technique used is based on a 3-phase protocol adapted from Giarratana et al.[28] The basic erythroid medium was IMDM (Life Technologies), 1 × LGlut-Pen-Strep, 10% BSA, 40 μg/mL inositol, 10 μg/mL folic acid, 1.6 μM monothioglycerol, 120 μg/mL transferrin, and 10 μg/mL insulin (all from Sigma-Aldrich, St. Louis, MO, USA). During the first phase (6 days), the cells were cultured in the presence of 1×10^{-6} M hydrocortisone (Sigma-Aldrich), 100 ng/mL hSCF, 5 ng/mL hIL-3 (Peprotech), and 3 IU/mL erythropoietin (Epo; Janssen Pharmaceuticals). In the second phase (3 days), the cells were transferred onto a stromal cell layer (MS-5, murine stromal cell line,[29] provided by Gay Crooks, UCLA, Los Angeles, CA, USA) with the addition of only Epo (3 IU/mL) to basic erythroid medium. At day 11, all the cytokines were removed from the medium, and the cells were co-cultured on the MS-5 stromal layer until day 14, when they were collected to extract genomic DNA and RNA.

Colony-Forming Unit Assay

Colony-forming unit (CFU) assays were performed using Methocult H4435 Enriched methylcellulose (Cat. no. 04445; StemCell Technologies, Vancouver, BC, Canada) according to the manufacturer's instructions, with minor modifications. Briefly, 25, 50, and 100 transduced $CD34^+$ cells were plated in duplicate into 35 mm gridded cell culture dishes. After 14 days of culture in 5% CO_2, 37°C, and a humidified atmosphere, the different types of hematopoietic colonies were identified and counted. CFUs were then plucked for genomic DNA isolation (NucleoSpin Tissue XS; Clontech Laboratories, Mountain View, CA, USA).

VCN Determination

For VCN determination, the Psi region of the LV genome assay was duplexed with the SDC4 endogenous reference gene. The Psi assay sequences were as follows: 5′-ACCTGAAAGCGAAAGGGAAAC-3′ (forward primer), 5′-CGCACCCATCTCTCTCCTTCT-3′ (reverse primer), and 5′-FAM-AGCTCTCTCGACGCAGGACTCGGC-31 ABFQ-3′ (probe) (Integrated DNA Technologies, San Diego, CA, USA). The SDC4 assay sequences were as follows: 5′-CAGGGTCTGG GAGCCAAGT-3′ (forward primer), 5′-GCACAGTGCTGGACATT GACA-3′ (reverse primer), and 5′-HEX-CCCACCGAACCCAAG AAACTAGAGGAGAAT-31ABFQ-3′ (probe) (Integrated DNA Technologies).

Reaction mixtures of 22 μL volume, comprising 1 × Droplet Digital PCR (ddPCR) Master Mix (Bio-Rad, Hercules, CA, USA), 400 nmol/L primers and 100 nmol/L probe for each set, 40 U DraI (New England

Biolabs, Ipswich, MA, USA) and 30–100 μg of the genomic DNA to study, were prepared and incubated at 37°C for 1 h. Droplet generation was performed as described in Hindson et al.[30] with 20 μL of each reaction mixture. The droplet emulsion was then transferred with a multichannel pipet to a 96-well propylene plate (Eppendorf, Hamburg, Germany), heat sealed with foil, and amplified in a conventional thermal cycler (T100; Bio-Rad). Thermal cycling conditions consisted of 95°C for 10 min, (94°C for 30 s and 60°C for 1 min; 55 cycles), 98°C for 10 min (1 cycle), and a 12°C hold. After PCR, the 96-well plate was transferred to a droplet reader (Bio-Rad). Acquisition and analysis of the ddPCR data were performed with the QuantaSoft software (Bio-Rad) provided with the droplet reader.

Determination of %βAS3-Globin mRNA by ddPCR

RT-PCR to detect %βAS3-globin mRNA/total β-globin transcript (% AS3) was performed as described in Urbinati et al.[31] Isolation of RNA was done using the RNeasy Plus Mini Kit (QIAGEN, Hilden, Germany), followed by reverse transcription, as described in the Invitrogen protocol (final concentration: 10 U/μL Moloney murine leukemia virus RT (M-MLV RT), 500 μmol/L deoxyribonucleotide triphosphates (dNTPs), 150 ng/μL random primers, 2 U/μL RNase OUT, 10 mmol/L DTT, and 1 × First-Strand buffer). Two TaqMan hydrolysis probes were used to obtain the ratio of (1) target (HBBAS3) over (2) reference (HBBTotal: all variants of endogenous β-globin–like mRNA transcripts) for the quantification of gene expression by ddPCR. Reaction mixtures and thermal cycling conditions were as described earlier. The assay sequences were as follows: 5′-GGA GAA GTC TGC CGT TAC TG-3′ (HBBAS3/Total F2), 5′-CAC TAA AGG CAC CGA GCA CT-3′ (HBBAS3/Total R2), 5′-FAM-ACA AGG TGA-ZEN-ACG TGG ATG CCG TTG-3′ Iowa Black FQ (HBBAS3 probe), 5′-HEX-AAC CTC TGG-ZEN-GTC CAA GGG TAG ACC AGC AG-3′ Iowa Black FQ (HBBTotal probe; Integrated DNA Technologies).

NSG Transplants

All animals involved in experiments were cared for and handled in accordance with protocols approved by the UCLA Animal Research Committee under the Division of Laboratory Medicine. Six- to twelve-week-old NSG mice were sub-lethally irradiated using a Cs-137 source at 250 Rad with a dose rate of approximately 100 Rad/min. Twenty-four hours after irradiation, 1×10^6 mPB $CD34^+$ cells (transduced in the presence of dmPGE2/Poloxamer synperonic F108 or vehicle control) were transplanted via retro-orbital injection. Female mice were used in all NSG xenograft experiments, to avoid sex-based variations in engraftment.

Analysis of NSG Xenografts for Engraftment and VCN

PB was collected from transplanted mice via retro-orbital puncture at 6 weeks. After euthanasia at 15 weeks, BM was isolated by crushing femurs and tibias with a mortar and pestle. PB and BM engraftment (expressed as the percentage of $hCD45^+$ cells of total $CD45^+$ [$hCD45^+mCD45^+$]) were determined by staining for mCD45-PE (30-F11), hCD45-APC (HI30), and DAPI. For VCN and lineage distribution analysis, $hCD45^+$ cells were enriched from BM using a bead-based selection kit (Miltenyi Biotec, Bergisch Gladbach, Germany). Human CD45-enriched cells were stained with Ghost 780 (viability dye; Tonbo Biosciences, San Diego, CA, USA) and the following antibodies: hCD45-FITC (HI30), mCD45-PE (30F11), hCD3-PerCp-Cy5.5 (UCHT1), hCD33-BV421 (WM53), hCD34-APC (581), and hCD19-PE-Cy7 (SJ25C1). All antibodies were purchased from BD Biosciences (San Jose, CA, USA). Genomic DNA was extracted from hCD45-enriched cells using the Purelink Genomic DNA Mini Kit (Invitrogen, Carlsbad, CA, USA) and analyzed for VCN as described above.

Statistical Analysis

Values are represented as the mean ± SD, unless stated otherwise. GraphPad Prism 6.0 (GraphPad Software, San Diego, CA, USA) was used for all statistical analyses. Statistical details of each experiment can be found in the figure legends, including the mean and error bars, numbers of replicates, statistical tests, and p values from comparative analyses that were performed. The p value was calculated with a confidence interval of 95% to indicate the statistical significance between groups. A p value < 0.05 was considered statistically significant.

SUPPLEMENTAL INFORMATION

Supplemental Information can be found online at https://doi.org/10.1016/j.omtm.2019.03.005.

AUTHOR CONTRIBUTIONS

Conceptualization, D.B.K., R.P.H., and B.C.-F.; Formal Analysis, K.E.M. and B.C.-F.; Investigation, K.E.M., K.O., R.X., and B.C.-F.; Writing – Original Draft, K.E.M.; Writing – Review & Editing, K.E.M., R.P.H., and D.B.K.; Visualization, K.E.M.; Supervision, D.B.K., B.C.-F., and R.P.H.; and Funding Acquisition, D.B.K.

CONFLICTS OF INTEREST

The authors declare no competing interests.

ACKNOWLEDGMENTS

The authors thank Devin Brown for his assistance in maintaining the mouse colonies. This work was supported by the California Institute for Regenerative Medicine (DR3-06945) and unrestricted funds from the UCLA Broad Stem Cell Research Center. K.E.M. was supported by the Whitcome Predoctoral Training Program at the UCLA Molecular Biology Institute and T32 Medical Scientist Training Program at UCLA (T32GM008042)

REFERENCES

1. Brousseau, D.C., Panepinto, J.A., Nimmer, M., and Hoffmann, R.G. (2010). The number of people with sickle-cell disease in the United States: national and state estimates. Am. J. Hematol. *85*, 77–78.
2. Modell, B., and Darlison, M. (2008). Global epidemiology of haemoglobin disorders and derived service indicators. Bull. World Health Organ. *86*, 480–487.
3. Madigan, C., and Malik, P. (2006). Pathophysiology and therapy for haemoglobinopathies. Part I: sickle cell disease. Expert Rev. Mol. Med. *8*, 1–23.
4. Morgan, R.A., Gray, D., Lomova, A., and Kohn, D.B. (2017). Hematopoietic Stem Cell Gene Therapy: Progress and Lessons Learned. Cell Stem Cell *21*, 574–590.

5. Romero, Z., Urbinati, F., Geiger, S., Cooper, A.R., Wherley, J., Kaufman, M.L., Hollis, R.P., de Assin, R.R., Senadheera, S., Sahagian, A., et al. (2013). β-globin gene transfer to human bone marrow for sickle cell disease. J. Clin. Invest. *123*, 3317–3330.

6. Negre, O., Bartholomae, C., Beuzard, Y., Cavazzana, M., Christiansen, L., Courne, C., Deichmann, A., Denaro, M., de Dreuzy, E., Finer, M., et al. (2015). Preclinical evaluation of efficacy and safety of an improved lentiviral vector for the treatment of β-thalassemia and sickle cell disease. Curr. Gene Ther. *15*, 64–81.

7. Kanter, J., Walters, M.C., Hsieh, M.M., Krishnamurti, L., Kwiatkowski, J., Kamble, R.T., von Kalle, C., Kuypers, F.A., Cavazzana, M., Leboulch, P., et al. (2016). Interim Results from a Phase 1/2 Clinical Study of Lentiglobin Gene Therapy for Severe Sickle Cell Disease. Blood *128*, 1176.

8. Heffner, G.C., Bonner, M., Christiansen, L., Pierciey, F.J., Campbell, D., Smurnyy, Y., Zhang, W., Hamel, A., Shaw, S., Lewis, G., et al. (2018). Prostaglandin E_2 Increases Lentiviral Vector Transduction Efficiency of Adult Human Hematopoietic Stem and Progenitor Cells. Mol. Ther. *26*, 320–328.

9. Hauber, I., Beschorner, N., Schrödel, S., Chemnitz, J., Kröger, N., Hauber, J., and Thirion, C. (2018). Improving Lentiviral Transduction of $CD34^+$ Hematopoietic Stem and Progenitor Cells. Hum. Gene Ther. Methods *29*, 104–113.

10. Cutler, C., Multani, P., Robbins, D., Kim, H.T., Le, T., Hoggatt, J., Pelus, L.M., Desponts, C., Chen, Y.B., Rezner, B., et al. (2013). Prostaglandin-modulated umbilical cord blood hematopoietic stem cell transplantation. Blood *122*, 3074–3081.

11. Fitzhugh, C.D., Hsieh, M.M., Bolan, C.D., Saenz, C., and Tisdale, J.F. (2009). Granulocyte colony-stimulating factor (G-CSF) administration in individuals with sickle cell disease: time for a moratorium? Cytotherapy *11*, 464–471.

12. Lagresle-Peyrou, C., Lefrère, F., Magrin, E., Ribeil, J.A., Romano, O., Weber, L., Magnani, A., Sadek, H., Plantier, C., Gabrion, A., et al. (2018). Plerixafor enables safe, rapid, efficient mobilization of hematopoietic stem cells in sickle cell disease patients after exchange transfusion. Haematologica *103*, 778–786.

13. Fenard, D., Ingrao, D., Seye, A., Buisset, J., Genries, S., Martin, S., Kichler, A., and Galy, A. (2013). Vectofusin-1, a new viral entry enhancer, strongly promotes lentiviral transduction of human hematopoietic stem cells. Mol. Ther. Nucleic Acids *2*, e90.

14. Wang, C.X., Sather, B.D., Wang, X., Adair, J., Khan, I., Singh, S., Lang, S., Adams, A., Curinga, G., Kiem, H.P., et al. (2014). Rapamycin relieves lentiviral vector transduction resistance in human and mouse hematopoietic stem cells. Blood *124*, 913–923.

15. Petrillo, C., Cesana, D., Piras, F., Bartolaccini, S., Naldini, L., Montini, E., and Kajaste-Rudnitski, A. (2015). Cyclosporin a and rapamycin relieve distinct lentiviral restriction blocks in hematopoietic stem and progenitor cells. Mol. Ther. *23*, 352–362.

16. Ngom, M., Imren, S., Maetzig, T., Adair, J.E., Knapp, D.J.H.F., Chagraoui, J., Fares, I., Bordeleau, M.E., Sauvageau, G., Leboulch, P., et al. (2018). UM171 Enhances Lentiviral Gene Transfer and Recovery of Primitive Human Hematopoietic Cells. Mol. Ther. Methods Clin. Dev. *10*, 156–164.

17. Lewis, G., Christiansen, L., McKenzie, J., Luo, M., Pasackow, E., Smurnyy, Y., Harrington, S., Gregory, P., Veres, G., Negre, O., and Bonner, M. (2018). Staurosporine Increases Lentiviral Vector Transduction Efficiency of Human Hematopoietic Stem and Progenitor Cells. Mol. Ther. Methods Clin. Dev. *9*, 313–322.

18. Petrillo, C., Thorne, L.G., Unali, G., Schiroli, G., Giordano, A.M.S., Piras, F., Cuccovillo, I., Petit, S.J., Ahsan, F., Noursadeghi, M., et al. (2018). Cyclosporine H Overcomes Innate Immune Restrictions to Improve Lentiviral Transduction and Gene Editing In Human Hematopoietic Stem Cells. Cell Stem Cell *23*, 820–832.e9.

19. Canté-Barrett, K., Mendes, R.D., Smits, W.K., van Helsdingen-van Wijk, Y.M., Pieters, R., and Meijerink, J.P.P. (2016). Lentiviral gene transfer into human and murine hematopoietic stem cells: size matters. BMC Res. Notes *9*, 312.

20. Höfig, I., Atkinson, M.J., Mall, S., Krackhardt, A.M., Thirion, C., and Anastasov, N. (2012). Poloxamer synperonic F108 improves cellular transduction with lentiviral vectors. J. Gene Med. *14*, 549–560.

21. Batrakova, E.V., Li, S., Vinogradov, S.V., Alakhov, V.Y., Miller, D.W., and Kabanov, A.V. (2001). Mechanism of pluronic effect on P-glycoprotein efflux system in blood-brain barrier: contributions of energy depletion and membrane fluidization. J. Pharmacol. Exp. Ther. *299*, 483–493.

22. Krylova, O.O., Melik-Nubarov, N.S., Badun, G.A., Ksenofontov, A.L., Menger, F.M., and Yaroslavov, A.A. (2003). Pluronic L61 accelerates flip-flop and transbilayer doxorubicin permeation. Chemistry *9*, 3930–3936.

23. Zonari, E., Desantis, G., Petrillo, C., Boccalatte, F.E., Lidonnici, M.R., Kajaste-Rudnitski, A., Aiuti, A., Ferrari, G., Naldini, L., and Gentner, B. (2017). Efficient Ex Vivo Engineering and Expansion of Highly Purified Human Hematopoietic Stem and Progenitor Cell Populations for Gene Therapy. Stem Cell Reports *8*, 977–990.

24. Ribeil, J.-A., Hacein-Bey-Abina, S., Payen, E., Magnani, A., Semeraro, M., Magrin, E., Caccavelli, L., Neven, B., Bourget, P., El Nemer, W., et al. (2017). Gene Therapy in a Patient with Sickle Cell Disease. N. Engl. J. Med. *376*, 848–855.

25. Thompson, A.A., Walters, M.C., Kwiatkowski, J., Rasko, J.E.J., Ribeil, J.A., Hongeng, S., Magrin, E., Schiller, G.J., Payen, E., Semeraro, M., et al. (2018). Gene Therapy in Patients with Transfusion-Dependent β-Thalassemia. N. Engl. J. Med. *378*, 1479–1493.

26. Zhang, D., Xu, C., Manwani, D., and Frenette, P.S. (2016). Neutrophils, platelets, and inflammatory pathways at the nexus of sickle cell disease pathophysiology. Blood *127*, 801–809.

27. Tisdale, J., Kanter, J., Mapara, M., Kwiatkowski, J., Krishnamurti, L., Schmidt, M., Miller, A.L., Pierciey, F.J., Shi, W., Ribeil, J.-A., et al. (2018). Current Results of Lentiglobin Gene Therapy in Patients with Severe Sickle Cell Disease Treated Under a Refined Protocol in the Phase 1 Hgb-206 Study. Blood *132*, 1026.

28. Giarratana, M.-C., Kobari, L., Lapillonne, H., Chalmers, D., Kiger, L., Cynober, T., Marden, M.C., Wajcman, H., and Douay, L. (2005). Ex vivo generation of fully mature human red blood cells from hematopoietic stem cells. Nat. Biotechnol. *23*, 69–74.

29. Suzuki, J., Fujita, J., Taniguchi, S., Sugimoto, K., and Mori, K.J. (1992). Characterization of murine hemopoietic-supportive (MS-1 and MS-5) and non-supportive (MS-K) cell lines. Leukemia *6*, 452–458.

30. Hindson, B.J., Ness, K.D., Masquelier, D.A., Belgrader, P., Heredia, N.J., Makarewicz, A.J., Bright, I.J., Lucero, M.Y., Hiddessen, A.L., Legler, T.C., et al. (2011). High-throughput droplet digital PCR system for absolute quantitation of DNA copy number. Anal. Chem. *83*, 8604–8610.

31. Urbinati, F., Wherley, J., Geiger, S., Fernandez, B.C., Kaufman, M.L., Cooper, A., Romero, Z., Marchioni, F., Reeves, L., Read, E., et al. (2017). Preclinical studies for a phase 1 clinical trial of autologous hematopoietic stem cell gene therapy for sickle cell disease. Cytotherapy *19*, 1096–1112.

OMTM, Volume *13*

Supplemental Information

PGE2 and Poloxamer Synperonic F108 Enhance Transduction of Human HSPCs with a β-Globin Lentiviral Vector

Katelyn E. Masiuk, Ruixue Zhang, Kyle Osborne, Roger P. Hollis, Beatriz Campo-Fernandez, and Donald B. Kohn

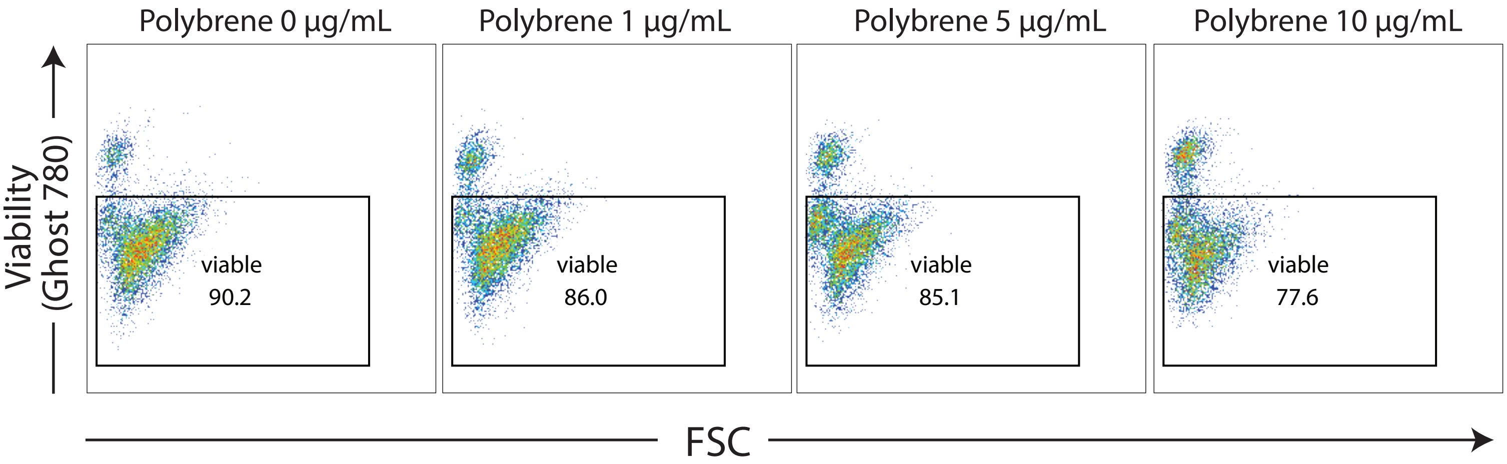

Figure S1: Dose-dependent toxicity of polybrene in LV transduction cultures

G-CSF mPB CD34+ cells were transduced with Globe1-AS3 in the presence of PGE2/poloxamer F-108 and increasing doses of polybrene. FACS plots show viability of cells measured 24 hours post-transduction

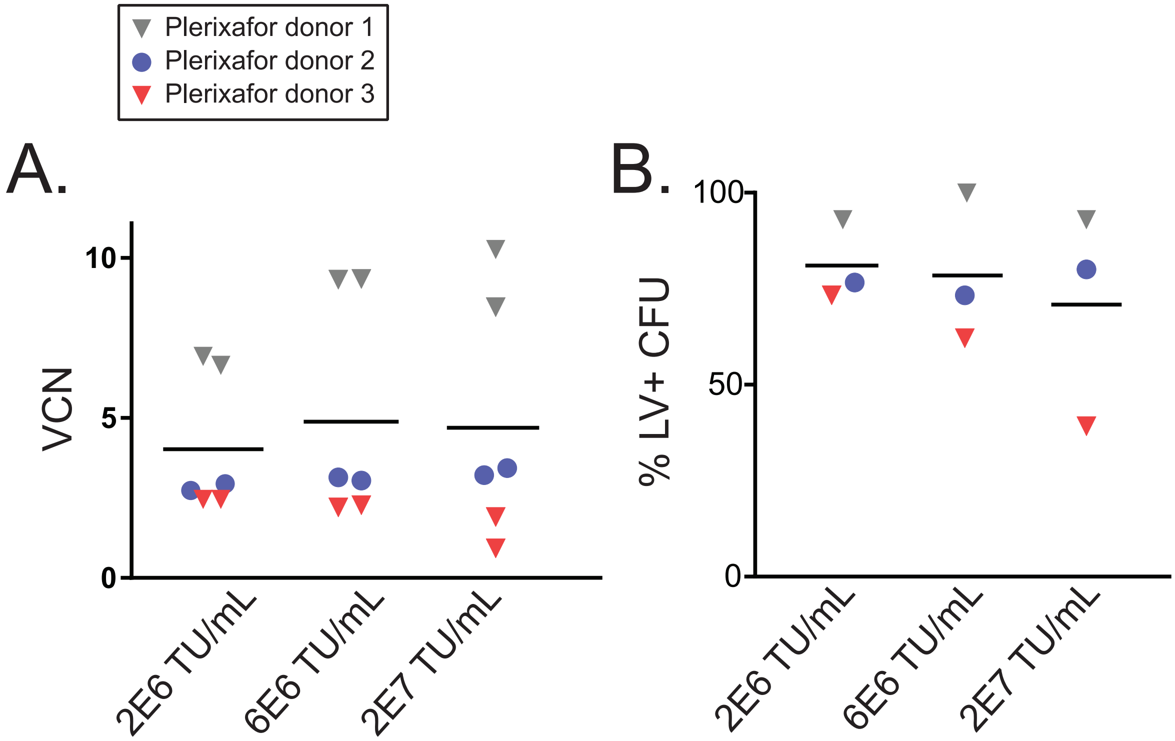

Figure S2: Transduction of Plerixafor-mobilized CD34+ cells with LV Globe1-AS3

A. VCN in 12 day myeloid differentiated cultures for Plerixafor mPB CD34+ cells transduced with transduction enhancers at 3 different LV doses. Data represent measurements from 2 replicate culture wells for 3 independent mPB CD34+ donors (each marked with a distinct color/symbol).
B. Percentage of individual colonies containing integrated viral copies for Plerixafor mPB CD34+ cells transduced with transduction enhancers at 3 different LV doses. A positive colony was defined as VCN>0.5. Data represent a single percentage (calculated from 30 analyzed colonies) for each LV dose in each of 3 independent CD34+ donors.

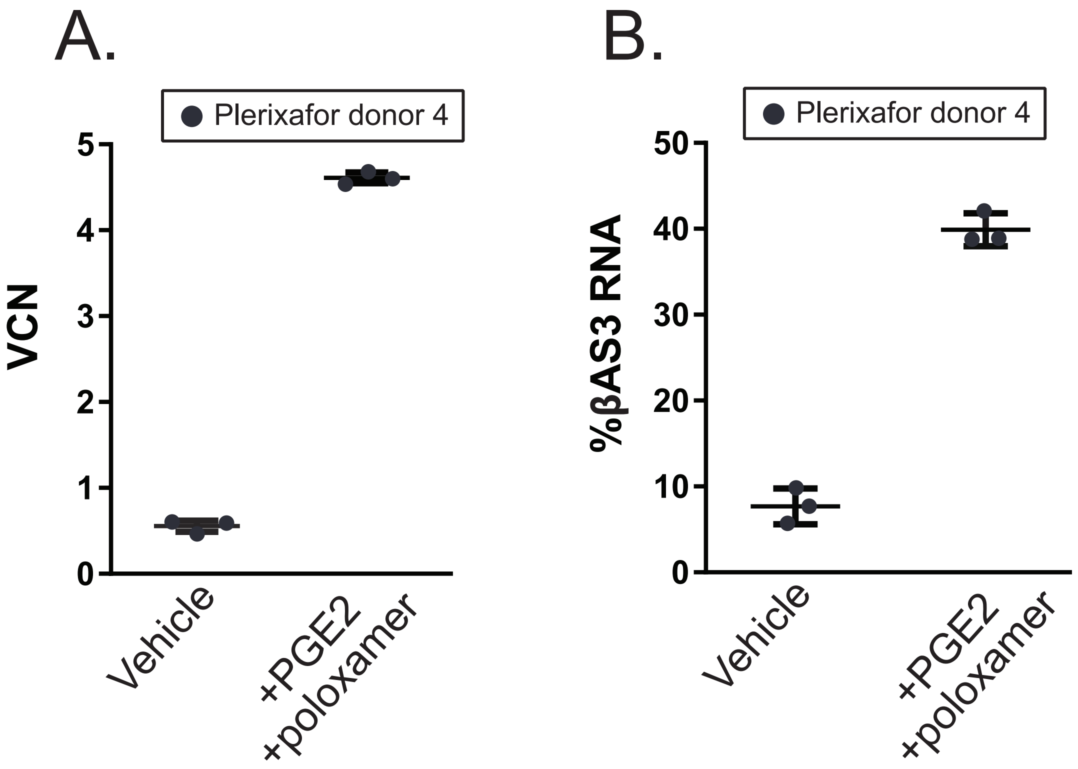

Figure S3: Correlation between enhanced VCN and enhanced βAS3 transgene expression

A. VCN in 12 day erythroid differentiated cultures for Plerixafor mPB CD34+ cells transduced with vehicle control or transduction enhancers at $2x10^6$ TU/mL. Data represent mean ± SD of 3 replicate culture wells from one CD34+ donor.

B. Corresponding percentage of viral βAS3 RNA (as a percentage of total β-globin RNA) measured in erythroid differentiated cells. Data represent mean ± SD of 3 replicate culture wells from one CD34+ donor.

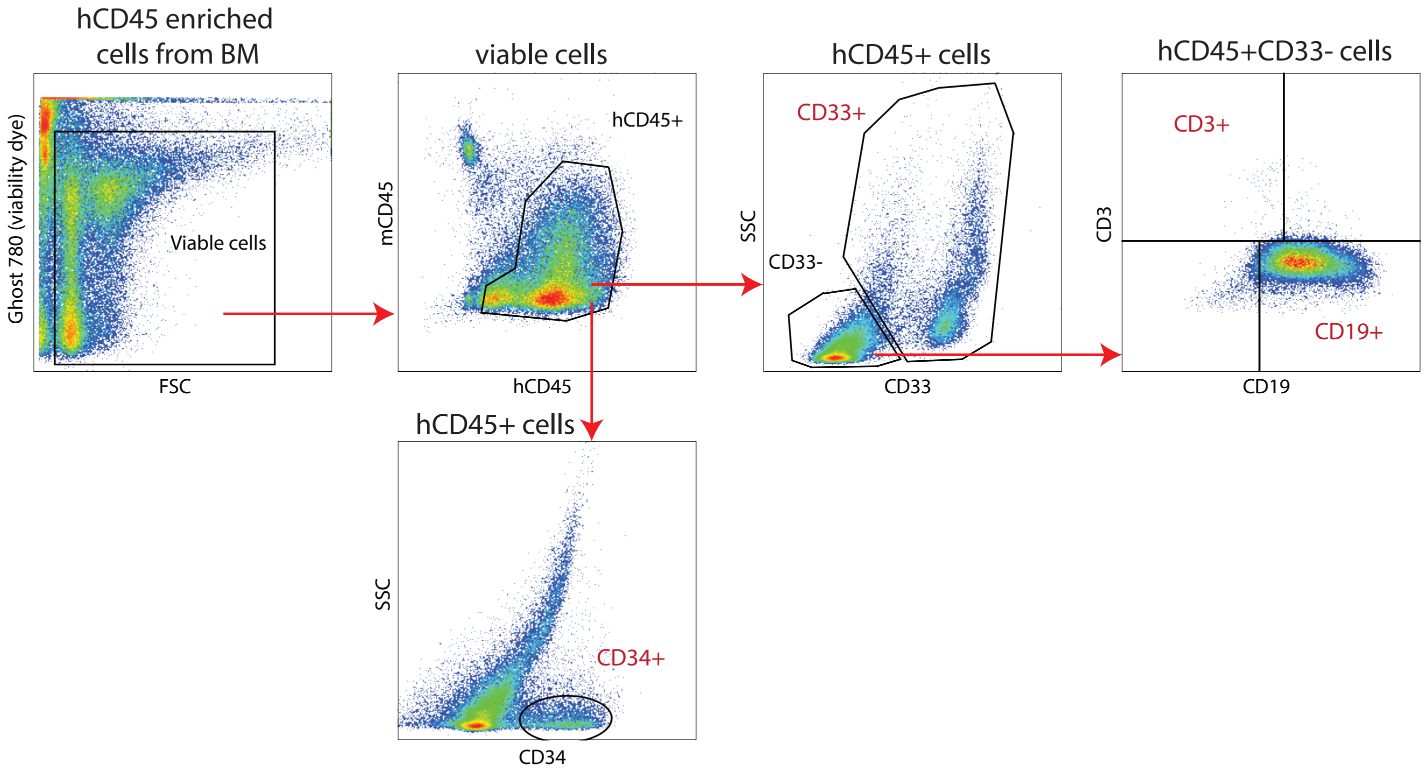

Figure S4: Determination of lineage distribution in NSG xenografts

Human CD45+ cells were magnetically enriched from the BM of engrafted NSG mice. Plots show flow cytometry gating strategy used to determine the relative contribution of each lineage to total engrafted hCD45+ cells. Lineages analyzed are marked in red and include: CD34+, CD33+, CD3+, and CD19+.
